# Innate immune crosstalk in ALS/FTD pathogenesis

**DOI:** 10.1016/j.cellin.2026.100340

**Published:** 2026-06-09

**Authors:** Xiaoqiu Shu, Xinyuan Yu, Pinglong Xu, Ailian Wang

**Affiliations:** a Sir Run Run Shaw Hospital, Zhejiang University School of Medicine, and Life Sciences Institute, Zhejiang University, Hangzhou, 310058, China; b Department of Hepatobiliary and Pancreatic Surgery and Zhejiang Provincial Key Laboratory of Pancreatic Disease, The First Affiliated Hospital, Zhejiang University School of Medicine, Hangzhou, 310058, China

**Keywords:** Amyotrophic lateral sclerosis, Frontotemporal dementia, cGAS-STING, NLRP3 inflammasome, TREM2, Microglia, Neuroinflammation

## Abstract

Marked by protein aggregation, impaired proteostasis, organelle stress, and chronic neuroinflammation, amyotrophic lateral sclerosis (ALS) and frontotemporal dementia (FTD) form a clinically, genetically, and pathologically overlapping disease spectrum. Increasing evidence indicates that innate immune activation is not merely a secondary response to neuronal injury, but an active driver of disease progression. In this review, we elaborate on how ALS/FTD-associated genetic lesions and pathogenic protein aggregates, including TDP-43, SOD1, FUS, and C9orf72-derived dipeptide repeat proteins, engage three interconnected innate immune pathways: cGAS-STING, NLRP3 inflammasomes, and TREM2-DAP12 signaling. We further highlight emerging crosstalk among these pathways, in which cGAS-STING and NLRP3 reinforce inflammatory signaling, while NLRP3-driven TREM2 shedding may impair microglial clearance and perpetuate proteostatic failure. Understanding this immune network may help define disease subtypes, identify biomarkers, and guide combinatorial therapeutic strategies that suppress harmful inflammation while preserving protective microglial functions.

## 1 Introduction

Amyotrophic lateral sclerosis (ALS) and frontotemporal dementia (FTD) belong to a spectrum of rapidly progressive and invariably fatal neurodegenerative disorders. ALS, a progressive and fatal neurodegenerative disorder, primarily targets motor neurons, leading to paralysis and respiratory failure within 3-5 years (Feldman et al., 2022). FTD, the second most common form of early-onset dementia, results in profound deterioration of behavior, personality, and language (Ng et al., 2015). Critically, these disorders exhibit significant clinical, genetic, and pathological overlap, with up to 50% of ALS patients exhibiting cognitive dysfunction and 15% of FTD patients developing motor symptoms, thereby blurring their diagnostic boundaries (Strong et al., 2009).

The pathogenic landscape of the ALS/FTD spectrum is complex and defined by two core hallmarks: the aberrant aggregation of specific proteins―such as SOD1, TAR DNA-binding protein 43 (TDP-43), FUS, and C9orf72-related dipeptide repeat proteins (DPRs) (Neumann et al., 2006; Wood et al., 2021), and the non-cell-autonomous activation of innate immunity. Genetic studies have been pivotal in underscoring the centrality of neuroinflammation and in identifying risk variants in genes highly expressed in microglia, including *TBK1*, *C9orf72*, and *TREM2*. These findings compellingly position innate immunity as an active driver rather than a passive bystander in disease pathogenesis.

Over the past decade, research has established that innate immune activation and the resulting neuroinflammation are not merely secondary responses but core pathological drivers of the ALS/FTD spectrum. Key innate immune pathways, such as cGAS-STING, NLRP3 inflammasome, and TREM2-DAP12, have been identified as critical contributors to this process. The cGAS-STING pathway detects aberrant cytosolic double-stranded DNA (dsDNA) to initiate type I interferon (IFN-I) signaling (Hopfner & Hornung, 2020; Ishikawa & Barber, 2008; Sun et al., 2009, 2013; Tan et al., 2022; Zhong et al., 2008), while the NLRP3 inflammasome integrates diverse danger signals to trigger the release of potent inflammatory cytokines (Deora et al., 2020). In contrast, the TREM2 receptor, identified as a genetic risk factor for neurodegeneration, orchestrates microglial responses and primarily promotes phagocytic clearance and tissue homeostasis (Shu et al., 2023, 2026). These systems coordinate a delicate balance between immune defense and regulatory control.

Microglia express pattern recognition receptors (PRRs) that can detect damage-associated molecular patterns (DAMPs). However, the precise functional impact of these interactions on disease progression remains an active area of investigation. Building upon this framework, the following sections detail how these key immune pathways sense dangerous protein aggregates and other DAMPs, driving the pathology of the ALS/FTD continuum.

## 2 ALS/FTD hallmarks: protein aggregates and innate immunity activation

**Fig. 1:**
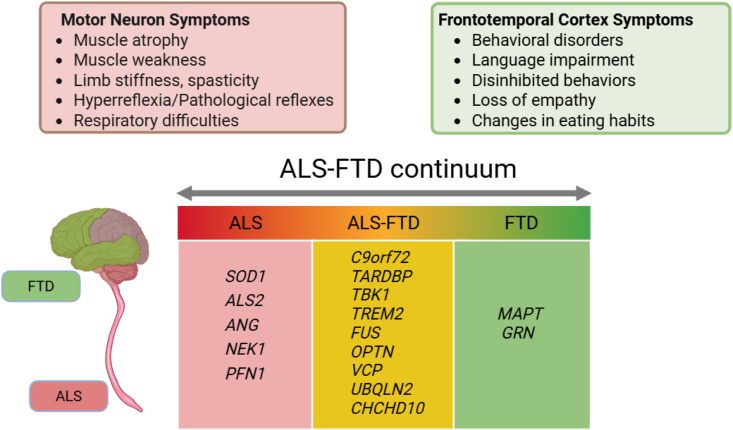
**Genetic landscape and clinical correlates across the ALS-FTD spectrum.** This figure illustrates the continuum from pure motor (ALS) to pure cognitive (FTD) presentations, with ALS-FTD representing an overlapping syndrome. The upper section outlines the primary clinical manifestations associated with motor neuron involvement (e.g., muscle weakness, atrophy, dysarthria) and frontotemporal cortical dysfunction (e.g., behavioral changes, executive deficits, language impairment). The lower table maps key causative genes onto this clinical spectrum. Genes such as *SOD1*, *ALS2*, and *NEK1* are predominantly linked to ALS, such as *C9orf72* and *TARDBP* are strongly associated with the ALS-FTD overlap. This schema highlights that genetic etiology correlates with, and potentially predicts, the predominant clinical phenotype along the ALS-FTD continuum.

The ALS and FTD disease spectrum is underpinned by shared risk genes and convergent pathological mechanisms, including *C9orf72*, *TARDBP* (which encodes TDP-43), *TBK1*, *FUS*, *OPTN*, *VCP*, *UBQLN2*, *CHCHD10*, and *TREM2*, which support the concept that the two disorders represent a disease continuum. The proteins encoded by these genes disrupt key cellular pathways including RNA metabolism, autophagy, immune responses, and vesicle dynamics (Balendra & Isaacs, 2018; Ling et al., 2013) (Fig. 1).

A common pathological hallmark is the progressive accumulation and spread of abnormal protein aggregates. These can arise spontaneously or from specific mutations, leading to SOD1 aggregates, TDP-43 inclusions, and C9orf72-related DPRs. These misfolded proteins and peptides accumulate in a characteristic spatiotemporal sequence within specific anatomical regions and propagate to interconnected brain areas. Cytoplasmic TDP-43 inclusions are a defining feature of both diseases, present in over 95% of ALS and approximately 50% of FTD cases, reflecting underlying disruptions in RNA metabolism and proteostasis (Wood et al., 2021). The C9orf72 hexanucleotide repeat expansion, the most common genetic cause of familial ALS (∼40%) and FTD (∼20%), promotes neurodegeneration through both loss-of-function and toxic gain-of-function mechanisms, including the formation of RNA foci and protein aggregates. Recent evidence provides decisive causal insight into this long-standing debate, demonstrating that selective suppression of repeat-associated non-AUG (RAN) translation abolishes DPR production while preserving RNA foci, and is sufficient to rescue neurodegeneration, behavioral deficits, and molecular pathology. These findings establish DPRs, rather than repeat RNAs, as the principal drivers of C9orf72-associated ALS/FTD pathogenesis (Jiang et al., 2026). Notably, inhibition of C9orf72-DPRs production also alleviates phosphorylated TDP-43 aggregation, suggesting that DPRs function upstream of TDP-43 pathology and may represent an initiating factor in disease progression (Jiang et al., 2026). Poly-Gly-Ala (poly-GA), the predominant DPR species in C9orf72-ALS/FTD, forms aggregation-prone structures that drive neurotoxicity and engage innate immune sensors (Renton et al., 2011; Zhang et al., 2021). The initiation of protein aggregation, along with associated synaptic and neuronal loss, begins in specific regions and spreads to other brain areas through mechanisms that remain under active investigation.

**Fig. 2:**
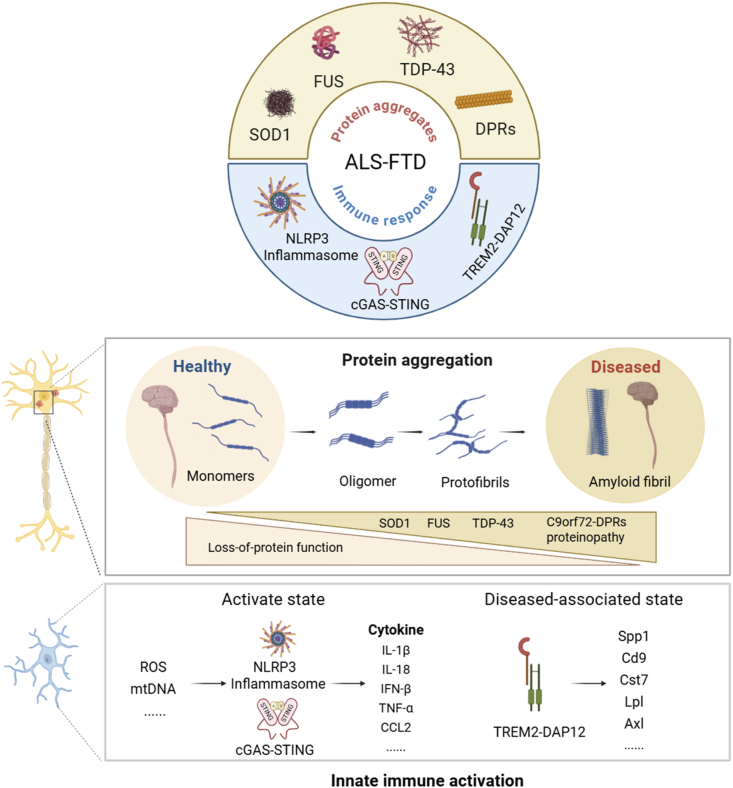
**Protein aggregation and innate immune activation in ALS/FTD.** Pathological aggregation of ALS/FTD-associated proteins drives the formation of toxic oligomers and fibrils. This process, together with cellular stressors such as reactive oxygen species (ROS) and mtDNA release, potently activates innate immune pathways, including the NLRP3 inflammasome and cGAS-STING signaling, triggering a pro-inflammatory cytokine response (e.g., IL-1β, IFNβ). In the diseased state, sustained activation signals through the TREM2-DAP12 axis critically promote the transition of microglia into a disease-associated state, characterized by markers such as Spp1, Axl and Cst7.

Innate immunity activation is another core hallmark of ALS/FTD, characterized by the chronic activation of resident immune cells in the central nervous system (CNS). In regions affected by neurodegeneration, these cells drive innate immune responses through PRRs that sense DAMPs. DAMPs include proteolytically cleaved extracellular matrix components, mitochondrial components (such as mitochondrial DNA (mtDNA) and ATP), and pathological protein aggregates, such as SOD1, TDP-43, and C9orf72-DPR. Supporting this concept, recent experimental evidence demonstrates that DPR accumulation is not only associated with cellular stress but is sufficient to drive neuroinflammatory responses, as suppression of DPR production significantly attenuates microglial activation and inflammatory signaling (Jiang et al., 2026). Within the ALS/FTD context, major PRR pathways play distinct roles: the NLRP3 inflammasome senses protein aggregates and releases interleukin-1β (IL-1β), while the cGAS-STING pathway detects aberrant cytosolic dsDNA to induce IFN-I, predominantly IFNβ. Both cytokine outputs intensify neuroinflammation and disease progression. In parallel, the TREM2-DAP12 complex regulates microglial engagement with pathological protein aggregates and neuronal debris (Fig. 2).

## 3 Innate immunity response and neuronal pathomechanisms

Innate immune responses mediated by CNS-resident cells are central drivers of pathology. Key ALS/FTD-associated mutations in genes such as *C9orf72*, *TBK1*, and *TREM2* converge on disrupted protein homeostasis and sustained neuroinflammation (McCauley & Baloh, 2019). The C9orf72 protein critically regulates the cGAS-STING pathway. Loss of C9orf72 function impairs STING degradation, thereby leading to its accumulation and hyperactivation of IFN-I responses (McCauley et al., 2020). Notably, emerging evidence further indicates that C9orf72-derived DPRs themselves act as potent upstream activators of STING signaling. Suppression of DPR production markedly reduces STING accumulation and downstream TBK1 phosphorylation, accompanied by attenuation of neuroinflammatory responses, thereby linking toxic protein species directly to innate immune activation (Jiang et al., 2026). TBK1, which functions both as a risk gene and a critical kinase effector within the cGAS-STING pathway, integrates nucleic acid sensing with inflammatory and autophagic responses (Liu et al., 2025; Brenner et al., 2019). Moreover, *TREM2* is a recognized risk gene in ALS/FTD (Cady et al., 2014; Guerreiro et al., 2013a); the TREM2 protein drives microglial survival, metabolic adaptation, and phagocytic activity, enabling the clearance of toxic aggregates such as poly-GA and TDP-43 (Shu et al., 2023; Xie et al., 2022). These protein inclusions can, in turn, activate the NLRP3 inflammasome, inducing pro-inflammatory factors like IL-1β that exacerbate the disease process.

**Fig. 3:**
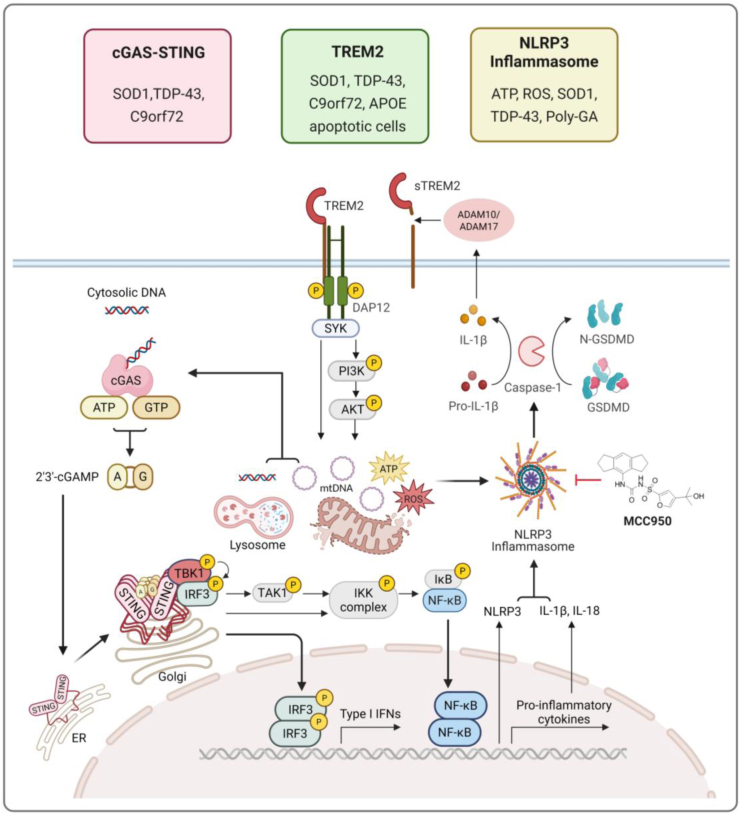
**DAMP sensing and innate immune signaling in ALS/FTD.** In ALS and FTD pathology, various DAMPs including aggregated proteins, ATP, cytosolic nucleic acids, and components of apoptotic cells activate specific PRRs. These include cell-surface receptors like TREM2, which binds ligands such as apoptotic cells, as well as intracellular sensors including cGAS (which detects cytosolic dsDNA) and the NLRP3 inflammasome (activated by ATP and ROS). Upon activation, these receptors initiate signaling cascades that converge in the nucleus to drive pro-inflammatory gene expression. This process typically involves the activation of transcription factors such as NF-κB and IRF3, resulting in the production of IFN-I and pro-inflammatory cytokines.

Collectively, the cGAS-STING pathway, the NLRP3 inflammasome, and the TREM2-DAP12 pathway intricately orchestrate neuroinflammation in ALS/FTD. In the following paragraphs, we will review immune signaling pathways and their specific functions in disease pathogenesis (Fig. 3).

### 3.1 cGAS-STING in ALS/FTD: from DNA sensing to neurodegeneration

The cGAS-STING pathway is a cornerstone of the innate immune system. cGAS, expressed in nearly all cell types, is a highly conserved cytosolic DNA sensor and functions as the primary detector of aberrant DNA. It detects various forms of dsDNA, ranging from pathogenic DNA derived from viruses and bacteria to aberrantly located self-DNA. These DNA species act as pathogen-associated molecular patterns (PAMPs) and DAMPs, respectively (Ma et al., 2024; Liu & Xu, 2025). Upon DNA binding, cGAS undergoes a conformational change and synthesizes the second messenger 2′3′-cyclic GMP-AMP (cGAMP). This molecule then binds to and activates the endoplasmic reticulum (ER)-resident adaptor protein STING. Activated STING translocates from the ER through the ER-Golgi intermediate compartment (ERGIC) to the Golgi apparatus. There, it serves as a platform for recruiting and activating TANK-binding kinase 1 (TBK1) and interferon regulatory factor 3 (IRF3). TBK1, the core kinase of the cGAS-STING pathway, directly phosphorylates IRF3, driving its nuclear translocation and the subsequent production of IFN-I. Importantly, TBK1 also acts as an essential conduit linking cGAS-STING to the NF-κB signaling. Specifically, TBK1 phosphorylates the IKK complex (primarily IKKβ), triggering IκBα degradation and NF-κB nuclear translocation. In parallel, activated STING engages TRAF6-dependent signaling to recruit transforming growth factor-β-activated kinase 1 (TAK1), which also converges on the IKK complex. Together, these TBK1- and TRAF6-mediated parallel cascades ensure robust canonical NF-κB activation and the induction of pro-inflammatory cytokines (Balka et al., 2020; Zhang et al., 2019). Ultimately, this canonical cGAS-STING network is crucial for mounting potent innate immune responses against viral infections, cellular stress, and tumors (Chen & Xu, 2023; Liu et al., 2025) (Fig. 3).

Beyond these canonical functions, several noncanonical cGAS-STING pathways have recently been elucidated, some of which are proposed to represent more primitive and conserved STING activities (Liu et al., 2025). The structural shift not only facilitates ER-to-Golgi trafficking of STING but also enables it to regulate organelle homeostasis and downstream signaling across different compartments. For instance, within the ER, STING directly stimulates the PKR-like endoplasmic reticulum kinase (PERK), regulating protein translation via the PERK-eIF2α axis (Zhang et al., 2022a). As it traffics through the ERGIC and post-Golgi vesicles, STING acts as a proton channel. This channel activity and the trafficking process itself are essential for initiating noncanonical autophagy via LC3 lipidation, activating transcription factor EB (TFEB) family members to promote lysosome biogenesis, and regulating Golgi cytokine transport (Gui et al., 2019; Lv et al., 2024; Poddar et al., 2025; Shang et al., 2019; Xun et al., 2024; Zhang et al., 2019).

Consequently, in the context of ALS and FTD, the cGAS-STING pathway has emerged as a central mediator of neuroinflammation, critically linking diverse pathological processes to innate immune activation. Although direct interactions between STING and ALS/FTD-associated pathological proteins remain unclear, dysregulated STING activation may indirectly drive proteostasis disruption associated with TDP-43 pathology and C9orf72-derived DPR toxicity. This disruption is exacerbated by STING-induced chronic ER stress, impaired vesicular trafficking, and lysosomal dysfunction (Freibaum et al., 2015; Lee et al., 2016; Zhang et al., 2018).

#### 3.1.1 cGAS-STING activation in ALS/FTD

The aberrant activation of cGAS-STING in ALS/FTD arises from multiple, convergent pathological triggers. *C9orf72* provides a key genetic link. The C9orf72 protein plays a crucial role in regulating the autolysosomal degradation of STING. In myeloid cells, C9orf72 deficiency impairs STING turnover, leading to its persistent activation and consequent hyperactivation of IFN-I responses. This mechanism establishes a state of innate immune autoinflammation, compellingly positioning the cGAS-STING pathway as an active driver of pathogenesis in this genetic subtype (McCauley et al., 2020). Mitochondrial dysfunction serves as another major trigger. Pathological protein aggregation, a hallmark of ALS/FTD, frequently induces mitochondrial damage. For instance, mitochondrial stress caused by mislocalized TDP-43 or mutant SOD1 promotes the release of mtDNA into the cytosol, thereby activating the cGAS-STING pathway (Tan et al., 2022; Yu et al., 2020). The mechanism for TDP-43 involves its entry into mitochondria, which facilitates mtDNA release through the mitochondrial permeability transition pore (mPTP) (Yu et al., 2020). Similarly, cytoplasmic aggregates of wild-type or mutant SOD1 induce mitochondrial dysfunction and facilitate the leakage of mtDNA and RNA-DNA hybrids, activating STING signaling (Tan et al., 2022).

Furthermore, while cGAS is canonically recognized as a cytosolic DNA sensor, its non-canonical nuclear functions provide a novel perspective on the neuronal genomic instability that characterizes ALS/FTD. TDP-43 pathology typically involves the loss of its normal nuclear function, leading to defective DNA repair and the progressive accumulation of DNA double-strand breaks (DSBs) (Mitra et al., 2019). Under these conditions of severe genomic stress, cGAS can translocate into the nucleus and bind directly to DSB sites. Strikingly, independent of its enzymatic activity, nuclear cGAS physically impedes the recruitment of homologous recombination (Liu et al., 2018; Zhao et al., 2020). Through this mechanism, nuclear cGAS is likely to synergistically exacerbate the DNA damage initially induced by TDP-43 pathology. The resulting amplified genomic instability generates more DNA fragments, which may subsequently leak into the cytosol, thereby driving a vicious, self-amplifying cycle of DNA damage and cGAS-STING-mediated neuroinflammation (Jiang et al., 2019).

Adding another layer of complexity, cGAS-STING may drive distinct interferon responses in ALS across species, potentially contributing to the varied disease manifestations observed. Studies show a conserved upregulation of interferon (IFN) genes and interferon-stimulated genes (ISGs) in both mouse and human ALS, with type I IFNs (IFNα/β) predominating in mouse models, whereas type II IFNs (IFNγ) signatures are more prominent in human samples (Hiew et al., 2025; Wang et al., 2011).

Notably, cGAS-STING activation in ALS/FTD is not confined to immune cells. Recent evidence demonstrates neuron-intrinsic STING activation in vulnerable motor neurons from human ALS post-mortem tissues and iPSC-derived models (Marques et al., 2024). This neuronal activation appears to be driven by DNA damage, positioning neurons not merely as passive victims but as active contributors to the neuroinflammatory environment in ALS/FTD. Persistent STING activation has also been observed in neurons from various lysosomal storage diseases (LSDs), in which lysosomal dysfunction leads to cytosolic dsDNA accumulation and cGAS-STING-driven neuronal death. This observation suggests a broader role for this pathway in neurodegeneration beyond ALS/FTD (Wang et al., 2024).

Therapeutically, targeting the cGAS-STING pathway holds considerable promise. STING inhibition suppresses the interferon response in C9orf72-ALS/FTD (McCauley et al., 2020). It also demonstrates efficacy in alleviating neuropathology and extending survival in both TDP-43 and SOD1 mutant models (Tan et al., 2022; Yu et al., 2020). The therapeutic relevance is further highlighted by findings that STING-TBK1 inhibition significantly attenuates neuronal damage in ALS models (Hiew et al., 2025). These findings collectively establish the cGAS-STING pathway as a pivotal node in ALS/FTD pathogenesis and a compelling therapeutic target across multiple genetic forms of the disease.

#### 3.1.2 TBK1 integrates the cGAS-STING signaling and autophagy

TBK1, the core kinase of the cGAS-STING pathway, plays complex, stage-dependent roles in ALS, functioning as a critical bridge between immune signaling and autophagy. In mouse models of ALS, heterozygous loss-of-function mutations in TBK1 impair autophagic clearance in motor neurons, thereby accelerating disease onset at early stages. Paradoxically, in later stages, the same deficiency can delay progression by significantly alleviating microglia-mediated neuroinflammation (Brenner et al., 2019). These contrasting effects emphasize the cell-type-specific and time-dependent complexity of TBK1's role in ALS pathology. Importantly, in C9orf72-ALS/FTD, the toxic dipeptide repeat protein poly-GA sequesters TBK1 within its intracellular inclusions, thereby impairing TBK1 kinase activity. In mice carrying TBK1 mutations, the pathogenic effects of TBK1 dysfunction and C9orf72-related pathology converge on disruption of the endosomal pathway, exacerbating TDP-43 aggregation and neurodegeneration. This observation indicates that C9orf72, TBK1, and TDP-43 interact via the endosomal pathway, integrating multiple pathogenic factors into a coherent disease mechanism (Shao et al., 2022).

TBK1's divergent roles across cell types highlight a critical layer of complexity in ALS pathogenesis. In neurons, TBK1 activity regulates autophagic clearance and endosomal trafficking, influencing early vulnerability and TDP-43 homeostasis. Conversely, in microglia, TBK1 deficiency alleviates neuroinflammation. These findings necessitate cell-targeted therapeutic strategies that account for TBK1's dynamic and context-dependent functions.

#### 3.1.3 Post-translational modifications of cGAS and STING: mechanistic intersections with ALS/FTD pathology

The intensity, duration, and precise subcellular localization of cGAS-STING signaling are tightly regulated by a complex network of post-translational modifications (PTMs). These PTMs act as molecular switches, and exploring how ALS/FTD-linked mutations disrupt these modifications provides crucial mechanistic insights into disease progression. Ubiquitination is central to determining STING's fate. While TRIM56-mediated K63-linked ubiquitination promotes STING oligomerization and recruitment of downstream kinases (Tsuchida et al., 2010), RNF5-catalyzed K48-linked polyubiquitination targets STING for proteasomal degradation (Zhong et al., 2009). Furthermore, palmitoylation at specific cysteine residues (Cys88 and Cys91 in human STING) is required for STING tetramerization and its successful trafficking from the ER to the Golgi (Mukai et al., 2016). Most critically, phosphorylation serves as the direct trigger for signal transduction. For instance, the phosphorylation of STING at Ser366 by TBK1 creates a docking site essential for IRF3 recruitment, leading to type I interferon production (Liu et al., 2015).

In the context of ALS/FTD, key genetic mutations directly intersect with these PTM networks. Loss-of-function mutations in *TBK1* directly impair its kinase activity, thereby abrogating the critical Ser366 phosphorylation of STING and disrupting downstream signaling (Freischmidt et al., 2015). Moreover, mutations in another risk gene *UBQLN2*, a protein associated with the ubiquitin-proteasome system, have been shown to significantly attenuate TBK1 auto-phosphorylation, highlighting how ALS-linked genetic defects can indirectly dampen or skew the PTM cascades (Chen et al., 2020). Future investigations into whether specific ALS mutations alter the balance of STING ubiquitination or its palmitoylation status will be vital for unraveling the precise immunopathological codes driving neurodegeneration.

### 3.2 NLRP3 inflammasome inflammatory response

The NOD-, LRR- and pyrin domain-containing 3 (NLRP3) inflammasome consists of the NLRP3 sensor protein, the adaptor protein ASC, and the effector caspase-1. As the most extensively studied inflammasome, the NLRP3 inflammasome is activated by a wide range of stimuli, including microbial PAMPs, host-derived DAMPs such as ATP and uric acid crystals, and environmental particulates (Lu et al., 2014). Its activation follows a characteristic two-step process. An initial priming signal, often from a Toll-like receptor (TLR), upregulates NLRP3 and pro-IL-1β transcription via NF-κB signaling, and induces post-translational modifications of NLRP3. A second activation signal then triggers NLRP3 oligomerization, leading to the helical assembly of ASC filaments. The resulting ASC speck acts as a platform for recruiting pro-caspase-1, which undergoes proximity-induced autocleavage to become active caspase-1. This enzyme subsequently processes the cytokines IL-1β and IL-18 into their mature forms (Swanson et al., 2019). In addition to this canonical pathway, a non-canonical NLRP3 pathway involves the direct activation of caspase-4/5/11 by cytosolic LPS, promoting pyroptosis through Gasdermin D (GSDMD) cleavage and the release of IL-1β (Kayagaki et al., 2015). Although the general principles of NLRP3 activation are understood, the specific second messengers that trigger its assembly remain a key area of ongoing research.

#### 3.2.1 NLRP3 signaling activation in disease progression

The NLRP3 inflammasome is a critical component of the innate immune system and an important mediator of neuroinflammation in the CNS, primarily within microglia, although these cells release less IL-1β than peripheral macrophages. Similar to its activation in other tissues, NLRP3 activation in the brain necessitates both priming and activation signals, culminating in inflammasome assembly and subsequent cytokine processing. Importantly, beyond classical DAMPs, other immune-stimulatory molecules present in the brain microenvironment, such as aggregated poly-GA and TDP-43, can also trigger NLRP3 activation (Shu et al., 2023; Zhao et al., 2015).

In the context of ALS, the mutant SOD1 protein (e.g., SOD1^G93A^) activates the NLRP3 inflammasome in microglia, in an ASC-dependent manner, leading to caspase-1 cleavage and IL-1β release. This age-dependent process is facilitated by the generation of ROS and peroxynitrite (Johann et al., 2015; Meissner et al., 2010). Cellular uptake of mutant SOD1 in its amyloid conformation is essential for this activation. Supporting its pathogenic role, genetic ablation of caspase-1 or IL-1β in SOD1^G93A^ mice prolongs survival, reduces glial activation, and mitigates spinal cord motor neuron loss. Similar protective effects are observed with the use of an anti-IL-1β antibody treatment (Deora et al., 2020; Johann et al., 2015). In human sporadic ALS, elevated levels of IL-18 and its binding protein in cerebrospinal fluid (CSF), along with increased expression of NLRP3, ASC, and active caspase-1 in spinal cord tissue, further corroborate NLRP3 inflammasome activation (Bellezza et al., 2018; Italiani et al., 2014).

ALS involves a dynamic pattern of innate immune activation. In murine models expressing mutant SOD1, this process begins in spinal motor neurons during early disease stages and later expands to become dominant within glial populations, particularly astrocytes. Human tissue analyses support this shift and are also observed in extra-motor regions such as the anterior thalamus, where they correlate with protein aggregation and neuronal dysfunction (Debye et al., 2018; Johann et al., 2015; Zhang et al., 2022b).

Therapeutic targeting of the NLRP3 inflammasome has proven challenging. The failure of interleukin-1 blockade to modify the disease course in patients suggests a pathophysiology that extends beyond a single cytokine axis, potentially involving alternative effectors such as IL-18 or pyroptotic cell death (Maier et al., 2015). This notion is supported by evidence that inhibiting a single component of this complex pathway is insufficient when multiple inflammatory platforms are engaged (Clenet et al., 2023). However, in C9orf72-mediated pathology, NLRP3 inhibition successfully restores microglial function and mitigates disease progression, indicating a viable therapeutic strategy for this genetic subtype (Shu et al., 2023).

#### 3.2.2 Pharmacological treatment with MCC950

MCC950 is a potent and selective small-molecule inhibitor of the NLRP3 inflammasome. It acts by directly binding to the NACHT domain of NLRP3, targeting a region adjacent to the Walker B motif, thereby blocking ATP hydrolysis—a critical step for NLRP3 activation (Coll et al., 2019). This binding stabilizes NLRP3 in an inactive, closed conformation, preventing its oligomerization and subsequent assembly with ASC and pro-caspase-1 into a functional inflammasome complex. As a result, MCC950 inhibits both canonical and non-canonical NLRP3 activation pathways, reducing the cleavage of pro-caspase-1 and the maturation and secretion of pro-inflammatory cytokines IL-1β and IL-18. Importantly, MCC950 does not affect other inflammasomes such as NLRP1, NLRC4, or AIM2, underscoring its specificity for NLRP3 (Coll et al., 2015). Its efficacy has been demonstrated across a wide range of inflammatory disease models.

MCC950 can penetrate the blood-brain barrier, making it a promising therapeutic candidate for central neurodegenerative diseases. In ALS models, MCC950 treatment in SOD1^G93A^ mice confirmed NLRP3 as the key inflammasome complex mediating microglial IL-1β secretion (Deora et al., 2020). In addition, pharmacological inhibition of NLRP3 by MCC950 in adult poly-GA mice reduced pathogenic poly-GA aggregates and alleviated motor and cognitive defects (Shu et al., 2023).

Nonetheless, the therapeutic impact of NLRP3 inhibition in ALS remains context-dependent, highlighting the complexity of its role within ALS-linked inflammatory networks. Although beneficial in some models, MCC950-mediated NLRP3 inactivation reduced the lifespan of SOD1^G93A^ mice (Moreno-Garcia et al., 2021). Moreover, acute NLRP3 inhibition failed to ameliorate spinal cord inflammation in the SOD1^G93A^ model, suggesting limited efficacy in a multi-inflammasome milieu (Clenet et al., 2023). These divergent outcomes underscore that while MCC950's ability to cross the blood-brain barrier is advantageous, its therapeutic application in ALS requires further investigation to clarify its role within the disease's complex inflammatory network.

### 3.3 TREM2 as a risk gene and regulator of microglial homeostasis

Triggering receptor expressed on myeloid cells 2 (TREM2) is a key regulator of microglial function, promoting cell survival, phagocytosis, and anti-inflammatory responses (Deczkowska et al., 2020; Yeh et al., 2017). These properties support its potential as a therapeutic target to enhance neuroprotective microglial activity in ALS/FTD. Current evidence implicates TREM2 in the clearance of pathogenic aggregates (Shu et al., 2023; Xie et al., 2022), and ongoing studies continue to clarify the roles of TREM2 genetic variants, soluble TREM2 (sTREM2), and downstream signaling pathways in disease pathogenesis. A deeper understanding of how TREM2 interacts with neurodegenerative mechanisms will be essential to exploit its therapeutic potential and modify disease progression in ALS/FTD.

#### 3.3.1 TREM2 expression in ALS/FTD

Neuroinflammation in ALS/FTD is primarily characterized by activation of innate immune-sensing pathways in microglia, the resident immune cells of the CNS. Microglia, indispensable guardians of brain homeostasis, exhibit a dual role in ALS/FTD pathogenesis, mediating both neuroprotective and neurotoxic effects. In early disease stages, microglia adopt a protective role, clearing cellular debris and protein aggregates (e.g., misfolded TDP-43) via phagocytosis (Spiller et al., 2018). However, chronic activation shifts them toward a pro-inflammatory state, marked by sustained release of cytotoxic factors like TNF-α, IL-1β, ROS, and nitric oxide (NO), which exacerbate neuronal damage (Henkel et al., 2009). Genetic mutations (e.g., *SOD1*, *C9orf72*) impair microglial lysosomal function, reducing clearance of toxic aggregates and amplifying neuroinflammation (Lall & Baloh, 2017; Xiao et al., 2007; Zhao et al., 2010). Post-mortem studies reveal activated microglia in ALS motor cortex, spinal cord, and FTD-associated frontal/temporal regions, correlating with neurodegeneration. Molecular imaging in ALS patients further confirms microglial activation that tracks with disease progression (Dols-Icardo et al., 2020).

TREM2, a microglial surface receptor, is essential for the induction and maintenance of the disease-associated microglia (DAM) phenotype, a reactive state pivotal to neurodegenerative disorders (Ulland & Colonna, 2018; Wang et al., 2025). DAM microglia transition from homeostatic surveillance to disease-specific repair state, balancing debris removal with inflammation control. TREM2 is essential for microglia to acquire a DAM profile fully, highlighting its critical role in neuroinflammatory responses. By sensing phospholipids and cellular debris via SYK-dependent signaling, TREM2 drives microglial survival, metabolic adaptation, and phagocytic activity, enabling clearance of toxic aggregates such as poly-GA in ALS (Shu et al., 2023; Wang et al., 2022). In FTD, TREM2 dysfunction correlates with synaptic degeneration and unchecked protein spread, accelerating cognitive decline (Borroni et al., 2014). Genetic TREM2 variants, like the ALS/FTD-linked R47H mutation, impair this transition, accelerating disease progression by reducing aggregate clearance, exacerbating neuroinflammation, and worsening motor neuron loss (Cady et al., 2014; Giannoccaro et al., 2017).

TREM2 expression is tightly regulated and varies across cell types and disease contexts. However, the molecular drivers underlying this regulation remain unclear. In neurodegenerative diseases, microglial TREM2 is upregulated to sustain DAM activation, thereby promoting phagocytosis of apoptotic cells and pathogenic proteins (Krasemann et al., 2017). In ALS, postmortem studies have revealed altered TREM2 mRNA and protein levels in the spinal cord, suggesting dysregulated microglial responses. TREM2 expression is elevated in the spinal cords of ALS patients and SOD1^G93A^ transgenic mice, indicating that TREM2 dysregulation is a feature of the disease. In human spinal cord tissue, TREM2 expression was upregulated in ALS patients, and this upregulation showed no association with other clinical features such as age at onset or site of onset (Cady et al., 2014). Ongoing research investigates the upregulation of sTREM2 in living ALS/FTD patients and correlates it with clinical outcomes (Jericó et al., 2023; Jiao et al., 2024; Woollacott et al., 2022).

Despite advances in understanding TREM2 in ALS, critical gaps remain in mapping its spatiotemporal expression dynamics, defining ligand specificity, and elucidating downstream signaling cascades. Addressing these questions is essential to unravel how TREM2-driven microglial adaptation shapes neurodegenerative processes, thereby guiding targeted therapeutic strategies tailored to disease-specific microglial phenotypes.

#### 3.3.2 TREM2 variants are associated with ALS/FTD

**Fig. 4:**
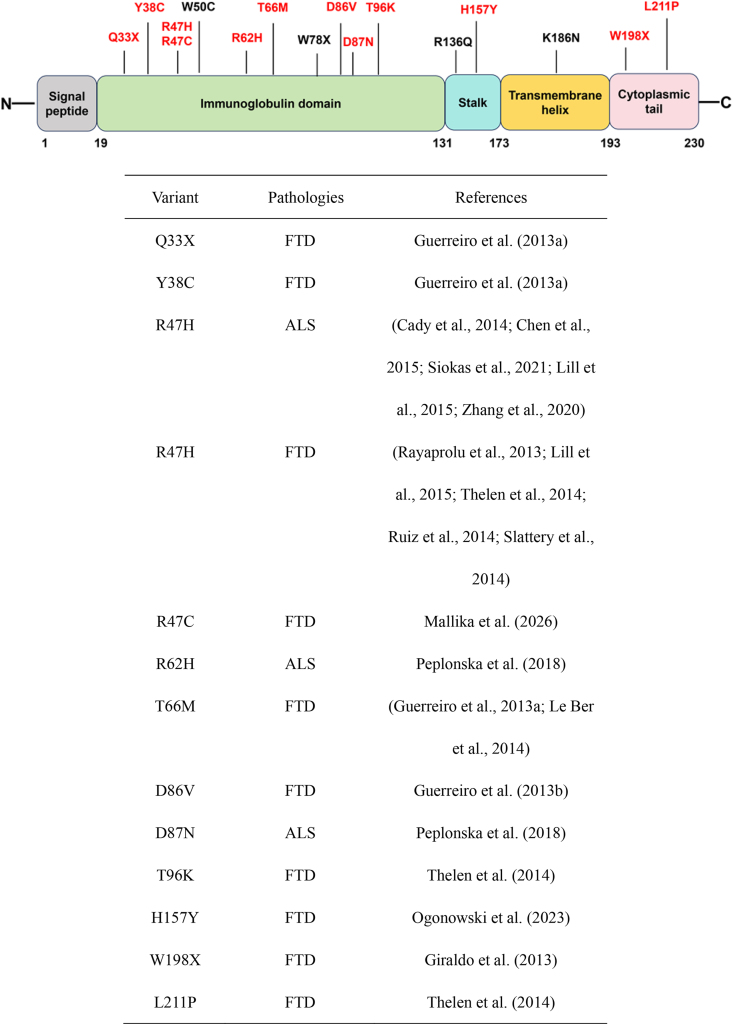
**Domains of TREM2 and disease-causing variants in ALS/FTD.** The schematic depicts the Ig-like domain, stalk, and transmembrane domain of TREM2. The table below summarizes TREM2 mutations that have been identified in patients with ALS/FTD, indicating their domain localizations and reported phenotypic associations (Giraldo et al., 2013, Guerreiro et al., 2013b, Le Ber et al., 2014, Zhang et al., 2020).

Although genome-wide association studies (GWAS) and rare variant analyses have explored the role of TREM2 in ALS and FTD, the findings remain less conclusive than its established links to Alzheimer's disease (Fig. 4). The TREM2 R47H variant has been implicated as a potential genetic risk factor for ALS, however its role remains controversial due to population-specific genetic heterogeneity (Chen et al., 2015; Rayaprolu et al., 2013; Siokas et al., 2021; Zhou et al., 2019). Early studies in cohorts linked this variant to sporadic ALS risk, proposing that its disruption of microglial phagocytosis and neuroinflammation—key ALS disease mechanisms—may drive pathogenesis (Cady et al., 2014). However, subsequent studies, including a large Chinese cohort analysis, failed to replicate this association, highlighting divergent genetic or environmental influences across populations (Chen et al., 2015). Meta-analyses pooling global data suggest only a modest overall risk effect, considerably weaker than its established associations with Alzheimer's and Parkinson's diseases (Lill et al., 2015). In addition to the R47H variant, rare TREM2 variants such as R62H and D87N have been identified in ALS patients. However, the mechanisms by which these variants contribute to the disease remain poorly characterized (Peplonska et al., 2018). Although conclusive genetic evidence is still lacking, TREM2's regulatory role in modulating neuroinflammation and myeloid cell function is consistent with established ALS mechanisms.

Studies have identified a positive association between TREM2 variants and FTD susceptibility, with specific variants such as R47H, T96K, and L211P demonstrating significant risk increases in specific cohorts (Borroni et al., 2014; Cuyvers et al., 2014; Rayaprolu et al., 2013; Ruiz et al., 2014; Thelen et al., 2014). Subsequent identification of a homozygous R47C variant in frontal variant Alzheimer's disease further expands the phenotypic spectrum of TREM2-associated neurodegeneration (Mallika et al., 2026). However, subsequent studies yielded conflicting results across populations (Lattante et al., 2013; Lill et al., 2015; Slattery et al., 2014; Thelen et al., 2014). Notably, while TREM2 variants may not universally elevate FTD risk in the general population, emerging evidence suggests they could modulate specific clinical or pathological endophenotypes of the disease, including reduced white matter volume, seizures, and motor symptoms (Baizabal-Carvallo & Jankovic, 2016).

TREM2 mutations (e.g., T66M, D86V) in Nasu-Hakola disease (NHD) manifest as early-onset behavioral variant frontotemporal dementia (bvFTD), with neuroimaging hallmarks such as callosal thinning, white matter lesions, and basal ganglia calcifications, often lacking bone cysts (Samanci et al., 2021). A homozygous TREM2 R47C mutation identified in a bvFTD patient (without bone involvement) was linked to reduced TREM2 expression, highlighting its atypical association with FTD (Ng et al., 2018). Heterozygous p. H157Y variants were found in three patients: two Colombian bvFTD cases with severe cognitive deficits and a Mexican-origin FTD-motor neuron disease case exhibiting TDP-43 pathology, suggesting region-specific neurodegeneration (Ogonowski et al., 2023). Recent studies underscore the role of TREM2 variants in FTD pathogenesis. Homozygous T66M mutations disrupt protein trafficking, reduce sTREM2 levels, and cause microglial dysfunction, impaired phagocytosis, and age-dependent metabolic decline (Kleinberger et al., 2017).

Emerging evidence suggests TREM2 variants contribute to clinical and phenotypic variability in FTD rather than serving as direct risk factors. Population-level uncertainty persists, reflecting differences in study cohorts, variant rarity, or underlying genetic architecture. Large-scale, multiethnic studies are imperative to clarify their broader contributions to FTD risk and subtype-specific pathogenic mechanisms.

#### 3.3.3 Integrated role of TREM2 in microglial regulation

Beyond its role in phagocytosis and protein aggregate clearance, TREM2 acts as a central regulator of microglial functional states in ALS/FTD. As summarized in our previous work (Shu et al., 2026), TREM2 orchestrates microglial fate transitions toward disease-associated phenotypes, while dynamically interacting with inflammatory signaling pathways such as the NLRP3 inflammasome and metabolic programs that govern energy utilization and stress responses. Notably, TREM2-dependent signaling can be either protective or detrimental depending on disease stage and pathological context, highlighting its role as a context-dependent modulator of neuroinflammation and neurodegeneration. Collectively, these findings position TREM2 as a key hub integrating phagocytic activity, inflammatory signaling, and metabolic adaptation in microglia during ALS/FTD progression.

#### 3.3.4 sTREM2 as a potential biomarker

sTREM2 is a cleaved fragment of the transmembrane receptor TREM2, generated through enzymatic cleavage of its extracellular domain. This process is mediated by ADAM10/17 metalloproteases, which cleave TREM2 at a specific site (H157-S158) near the cell membrane (Feuerbach et al., 2017; Kleinberger et al., 2014; Wunderlich et al., 2013). Following shedding, sTREM2 is released into biofluids such as CSF, while γ-secretase further processes the remaining membrane-bound fragment into a smaller intracellular domain with unclear roles (Wunderlich et al., 2013). Functionally, sTREM2 is not merely a byproduct but functions as a dynamic regulator of microglial activity. Initially, TREM2 was thought to function primarily as a decoy receptor that competed with full-length TREM2 for ligand binding. However, recent findings reveal that it also plays distinct roles in supporting microglial survival, enhancing inflammatory responses, and regulating interactions with DAMPs such as lipids and apoptotic debris (Zhong et al., 2017, 2019). Therefore, its levels in CSF reflect microglial activation states, making it a biomarker for neurodegenerative disease progression (Jiao et al., 2024; Suárez-Calvet et al., 2016).

sTREM2 is elevated in CSF and serum of ALS patients, correlating with upper motor neuron burden, disease progression, and axonal injury markers like neurofilament light chain. These associations are stronger in patients with disease duration >12 months, suggesting chronic neuroinflammatory involvement (Jiao et al., 2024). Studies show increased levels of TREM2 mRNA and protein in ALS spinal cord microglia, consistent with its role in modulating neuroinflammation and phagocytic clearance of pathological TDP-43 aggregates. CSF sTREM2 levels correlate with phosphorylated TDP-43 and predict survival in late-stage disease, while serum sTREM2 distinguishes ALS from controls (Jericó et al., 2023). Early sTREM2 elevation may reflect adaptive microglial responses to neurodegeneration, with sustained levels linked to prolonged survival (Cooper-Knock et al., 2017). These findings position sTREM2 as a promising biomarker for monitoring ALS progression and UMN pathology, with implications for therapeutic targeting. Notably, plasma sTREM2 levels are also elevated in non-Alzheimer's dementias including FTD, particularly in amyloid-negative and tau-negative subgroups, suggesting that peripheral sTREM2 elevation can occur independently of classical AD pathology (Guven et al., 2026).

CSF sTREM2 exhibits marked variability across FTD subtypes, reflecting heterogeneous microglial responses. While studies report no consistent elevation in *CHMP2B*- or *GRN*-associated FTD, or *C9orf72* and *MAPT*-linked cases, intra-group variability hints at dynamic microglial activity in mutation carriers (Kleinberger et al., 2014; van der Ende et al., 2021; Woollacott et al., 2022). In contrast, non- Alzheimer's tauopathies show elevated sTREM2, suggesting distinct pathological mechanisms (Rauchmann et al., 2020; Zhong et al., 2019). A consistent correlation between sTREM2 and tau biomarkers (p-tau, total tau) across FTD and control groups suggests microglial involvement in neurodegeneration. Variability may arise from spatiotemporal microglial dynamics, disease stage, or genetic factors. Notably, homozygous TREM2 mutations (T66M, Y38C) cause drastically reduced CSF sTREM2 due to loss of function (Kleinberger et al., 2014). Conversely, higher sTREM2 in presymptomatic *GRN* carriers correlates with slower progression (van der Ende et al., 2021).

These pathways have been extensively studied across a wide range of experimental models and patient-derived samples, highlighting their cell-type-specific activities and, as will be discussed in the next section, their intricate interconnections. Table 1 summarizes the key experimental evidence, detailing the diverse disease models, major cellular targets, and current therapeutic strategies aimed at modulating these pathways. This comprehensive overview underscores the mechanistic importance of neuroinflammation in ALS/FTD and lays the foundation for developing multifaceted therapeutic approaches.

**Table 1:** Experimental evidence for cGAS-STING, NLRP3, and TREM2 pathways in ALS/FTD.

Pathway	Models	Main cell types	Direct effects		Therapeutic strategies	Refs
cGAS-STING	*C9orf72* deficiency	Myeloid cells	STING dependent inflammatory response		STING inhibitor H151	McCauley et al. (2020)
	C9orf72-ALS/FTD mice	Motor neurons			DPR synthesis disruption	Jiang et al. (2026)
		Motor neurons			cGAS inhibitor RU.521; STING inhibitor H151	Marques et al. (2024)
		Motor neurons	loss of TBK1 activity and endosomal defects		/	Shao et al. (2022)
	Prp-TDP-43^Tg/+^ mice	Motor neurons	STING dependent inflammatory response		Genetic Deletion of *Sting*; STING inhibitor H151	Yu et al. (2020)
	ALS-SOD1 models (SOD1 ^G85R^/SOD1^G93A^)	Microglia, astrocytes			cGAS inhibitor RU.521; STING inhibitor H151 and C-176	Tan et al. (2022)
	SOD1^G93A^ mice	Microglia, astrocytes			siRNA-mediated knockdown of STING or TBK1; STAT1 inhibitor fludarabine	Hiew et al. (2025)
	Heterozygous *Tbk1* loss in SOD1^G93A^ mice	Motor neurons, microglia, astrocytes	Early stage: TBK1 dependent neuronal autophagy impairment	Late stage: reduced glial cell inflammatory response	/	Brenner et al. (2019)
NLRP3	SOD1^G93A^ mice; TDP-43^Q331K^ mice	Microglia	NLRP3 inflammasome activation; caspase-1 activation and IL-1β secretion		NLRP3 inhibitor MCC950	Deora et al. (2020)
	SOD1^G93A^ mice	Ventral horn neurons, microglia, astrocytes			/	Zhang et al. (2022b)
		Spinal cord astrocytes				Johann et al. (2015)
		Glial cells			17β-estradiol (E2)	Heitzer et al. (2017)
		Astrocytes and neurons	Expression of NLRP3 inflammasome components and IL-1β		/	Debye et al. (2018)
		Skeletal muscle	Increased NLRP3 inflammasome gene and protein levels			Moreno-Garcia et al. (2021)
	The wobbler mice	Neurons, microglia, astrocytes	NLRP3 activation; cleavage of GSDMD and pore formation			Cihankaya et al. (2024)
	SOD1^G93A^ mice; SOD1-mutant iPSC-derived microglia	Microglia	Release of IL-1β, IL-1α and TNF-α		Genetic Deletion of *Nlrp3*; NLRP3 inhibitor MCC950	Clenet et al. (2023)
	32 ALS patients	Plasma	Decreased miR-223 expression; increased NLRP3 and IL-1β levels		/	Dezfouli et al. (2025)
	Poly-PR-treated microglia	Microglia	NLRP3 inflammasome activation; caspase-1 activation and IL-1β secretion		NLRP3 inhibitor MCC950	Fu et al. (2022)
NLRP3/TREM2	C9orf72 poly-GA model	Microglia	Activation of the NLRP3 inflammasome promotes ADAM10-mediated TREM2 cleavage		NLRP3 inhibitor MCC950	Shu et al. (2023)
TREM2	SOD1^G93A^ mice	Disease-associated microglia (DAM)	DAM are activated sequentially by Trem2-independent and -dependent pathways		/	Keren-Shaul et al. (2017)
	rNLS8 TDP-43 mice	Microglia	TREM2-DAP12 axis promotes rod-shaped microglia and neuroprotection			Xie et al. (2025)
	rNLS8 TDP-43 mice; AAV-hTDP-43 model	Microglia	TDP-43 is a possible ligand for microglial TREM2 and that this interaction mediates neuroprotection of microglia in TDP-43-related neurodegeneration			Xie et al. (2022)
	SOD1^G93A^ mice	Microglia	The TREM2-APOE pathway regulates neurodegenerative microglial phenotypic switch		Genetic Targeting of *Trem2*	Krasemann et al. (2017)

## 4 NLRP3 serves as an important node linking the cGAS-STING and TREM2 pathways

Beyond their individual roles, the NLRP3 inflammasome, cGAS-STING pathway, and TREM2 receptor form a highly interconnected signaling network that critically influences microglial responses and drives disease progression in ALS/FTD. The cross-talk between these pathways generates a self-reinforcing cycle of neuroinflammation and disrupted proteostasis.

The cGAS-STING pathway and the NLRP3 inflammasome exhibit synergistic activation, particularly in response to mitochondrial dysfunction. Cytosolic mtDNA, released through channels such as the voltage-dependent anion channel (VDAC), serves as a common upstream trigger. This mtDNA is directly recognized by cGAS, initiating STING-dependent IFN-I production, while oxidized mtDNA fragments can specifically induce NLRP3 inflammasome assembly, culminating in caspase-1-dependent maturation of IL-1β and IL-18 (Xian et al., 2022). These pathways form a feed-forward inflammatory loop, where STING activation amplifies NLRP3-mediated responses, and the inflammatory cytokines produced by NLRP3 further sustain the cGAS-STING pathway. The therapeutic significance of this cross-talk is reflected in evidence showing that inhibition of the cGAS-STING pathway or blocking mPTP opening significantly reduces NLRP3 activation and neuronal damage in neurodegeneration models (Xian et al., 2022; Zhang et al., 2022c).

Beyond mtDNA-dependent mechanisms, STING activation may promote NLRP3 inflammasome assembly through additional organelle stress pathways. Dysregulated STING signaling can perturb intracellular homeostasis and organelle function, which may contribute to lysosomal stress and facilitate the release of lysosomal contents, including cathepsins. Lysosomal membrane destabilization and cathepsin release represent important upstream signals that promote NLRP3 inflammasome activation, leading to ASC recruitment, caspase-1 activation, and subsequent maturation of IL-1β and IL-18 (Broz & Dixit, 2016; Hornung et al., 2008; Nguyen et al., 2025). In parallel, disruption of ionic homeostasis, particularly potassium efflux, acts as a conserved trigger for NLRP3 oligomerization and inflammasome assembly (Broz & Dixit, 2016; Munoz-Planillo et al., 2013). Therefore, STING-associated organelle stress and metabolic perturbation may provide additional mechanisms integrating cGAS-STING signaling with NLRP3 inflammasome activation beyond mtDNA sensing.

A pivotal interface in this network involves NLRP3-mediated negative regulation of TREM2. In C9orf72-ALS models, poly-GA aggregates activate the NLRP3 inflammasome, driving IL-1β production and inducing ADAM10-dependent cleavage of TREM2 (Shu et al., 2023). This shedding process results in the generation of sTREM2 while depleting full-length, functional TREM2 from the microglial surface, thereby diminishing microglial phagocytic capacity and impairing aggregate clearance. The consequent accumulation of pathological proteins perpetuates NLRP3 activation, creating a vicious cycle of inflammation and proteostasis failure.

**Fig. 5:**
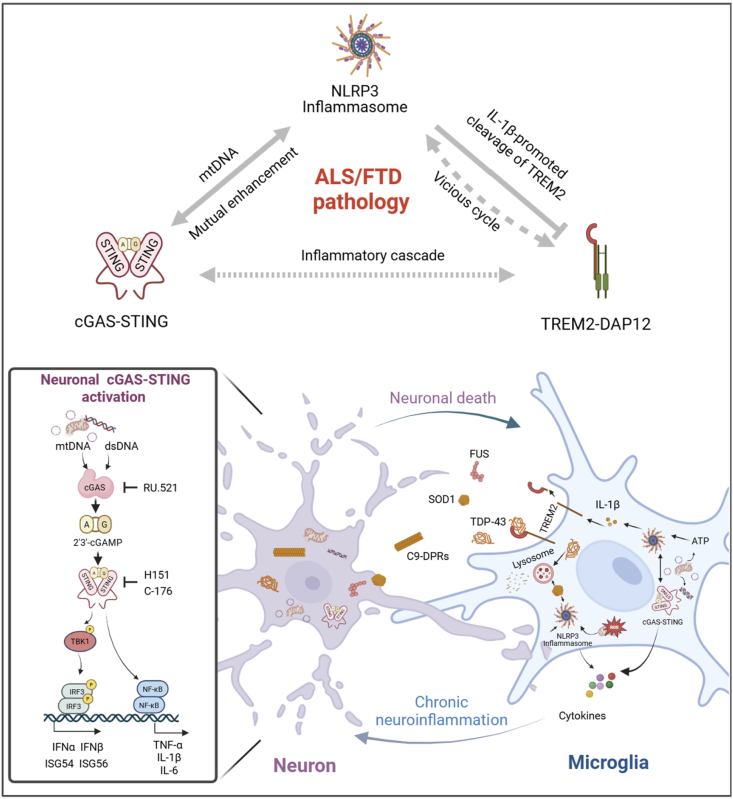
**Crosstalk among NLRP3 inflammasome, cGAS-STING, and TREM2.** Upon sensing neuronal protein aggregates, microglial cGAS-STING and NLRP3 inflammasome pathways are activated, leading to the production of pro-inflammatory cytokines, including IL-1β, TNF-α, and IFNβ. IL-1β promotes ADAM10/17-mediated cleavage of TREM2, impairing its phagocytic function and reducing clearance of pathological protein inclusions. The resulting accumulation of aggregates further potentiates NLRP3 and cGAS-STING activation, establishing a vicious cycle of chronic neuroinflammation. This sustained inflammatory milieu ultimately drives motor neuron dysfunction and death, highlighting the critical interplay between innate immune activation and proteostasis loss in disease progression.

The interplay between cGAS-STING and TREM2 in ALS/FTD represents an emerging area of investigation, with current understanding largely inferred from related models. On one hand, chronic activation of cGAS-STING signaling, driven by IFN-I and NF-κB, can induce extensive inflammatory reprogramming that suppresses microglial phagocytic pathways. This observation provides a potential mechanism by which hyperactivity of the cGAS-STING pathway indirectly inhibits TREM2-driven clearance of aggregates like TDP-43, effectively linking nucleic acid-sensing inflammation to proteostasis failure (Udeochu et al., 2023). On the other hand, the possibility of TREM2 dysfunction feeding back into the inflammatory cycle remains speculative. While direct evidence in ALS/FTD is lacking, insights from Alzheimer's disease models suggest that the TREM2 R47H mutation may impair mitochondrial homeostasis, leading to mtDNA leakage and further stimulating the cGAS-STING pathway (Carling et al., 2024). This observation raises the possibility of a bidirectional relationship between these pathways, forming a shared inflammatory loop that accelerates disease progression. Taken together, these pathways position NLRP3 as a central inflammatory hub that can amplify cGAS-STING signaling while simultaneously suppressing TREM2-mediated protective functions. This triad constitutes an integrated network in which mitochondrial stress orchestrates the parallel activation of cGAS-STING and NLRP3, both of which collectively inhibit TREM2-dependent clearance mechanisms (Fig. 5).

## 5 Conclusion and perspectives

Innate immune activation is a central hallmark of ALS and FTD, intimately linked to core pathological processes including protein aggregation, synaptic dysfunction, and neuronal death. The persistent and heightened activation of innate immune pathways such as the NLRP3 inflammasome and cGAS-STING promotes the seeding, spread, and accumulation of misfolded proteins. In contrast, TREM2 enhances microglial phagocytosis and clearance of these aggregates, thereby exerting neuroprotective effects and rescuing motor and cognitive functions in ALS/FTD models.

The NLRP3 inflammasome serves as a key inflammatory hub, responding to pathogenic proteins such as SOD1, TDP-43, and poly-GA. It engages in extensive cross-talk with other immune pathways: it can amplify cGAS-STING signaling and IFN-I responses, while also negatively regulating TREM2-mediated phagocytosis through ADAM10-mediated cleavage. This review positions NLRP3 at the center of a self-reinforcing cycle of neuroinflammation and impaired proteostasis. Consequently, pharmacological inhibition of NLRP3 (e.g., with MCC950) has shown promise in certain models, such as mitigating pathology in poly-GA mice. However, the outcomes are complex and context-dependent, underscoring the need for precise therapeutic targeting.

Aberrant activation of the cGAS-STING pathway emerges from multiple, independent pathological processes in ALS/FTD. C9orf72 deficiency in myeloid cells disrupts autolysosomal degradation of STING, thereby triggering persistent STING activation and the subsequent autoinflammatory response. Separately, TDP-43-induced mitochondrial dysfunction leads to the release of mtDNA, engaging the cGAS-STING axis upstream. This convergence of disparate disease initiators on the cGAS-STING pathway underscores its role as a critical node in neuroinflammation. Furthermore, the key kinase TBK1 within this pathway exhibits stage-dependent functions, underscoring the intricate and dynamic role of cGAS-STING in disease pathogenesis.

In contrast to the predominantly detrimental roles of NLRP3 and cGAS-STING, TREM2-DAP12 activation generally promotes microglial phagocytosis, aggregate clearance, and neuronal survival, although its effects may be context-dependent. However, its function can be compromised by NLRP3-mediated cleavage and its activation may occur at a later disease stage in ALS compared to other neurodegenerative diseases. The complexity of TREM2 biology is further reflected in the variable performance of sTREM2 as a biomarker and the challenges faced by TREM2-targeted therapies in clinical trials.

Significant challenges characterize the future of this field:

1) Combination and precise therapeutic strategies: Future interventions may need to move beyond single-pathway inhibition. Strategies that simultaneously dampen detrimental pathways (e.g., NLRP3, cGAS-STING) while bolstering protective mechanisms (e.g., TREM2) represent a promising direction. Precision targeting of critical integrators, such as TBK1, also holds promise but requires context-specific application.

2) Biomarker development and patient stratification: Defining the temporal dynamics of innate immune activation and validating biomarkers such as cGAMP and sTREM2 in large, longitudinal cohorts are essential for patient stratification and monitoring therapeutic efficacy.

3) Optimization of therapeutic agents: The mixed outcomes of NLRP3 inhibition, the complex role of TBK1, and setbacks in TREM2-based therapies highlight the importance of timing, context, and off-target effects. Developing agents with improved blood-brain barrier penetration and cell-type specificity remains a priority.

In summary, the intricate interplay among NLRP3 inflammasome, cGAS-STING, and TREM2-DAP12 fundamentally shapes the pathogenesis of ALS and FTD, with TBK1 emerging as a pivotal molecule linking innate immunity with autophagic degradation. Deciphering this complex immune network will be essential for developing the next generation of effective immunomodulatory therapies capable of halting or slowing these devastating diseases.

## CRediT authorship contribution statement

**Xiaoqiu Shu:** Writing – review & editing, Writing – original draft, Investigation, Conceptualization. **Xinyuan Yu:** Writing – review & editing, Visualization, Investigation. **Pinglong Xu:** Writing – review & editing, Funding acquisition, Conceptualization. **Ailian Wang:** Writing – review & editing, Supervision, Investigation, Funding acquisition, Conceptualization.

## Declaration of competing interest

The authors declare that they have no known competing financial interests or personal relationships that could have appeared to influence the work reported in this paper.

## Figures and Tables

**Table:** 

ADAM10	A disintegrin and metalloproteinase domain10
ALS	Amyotrophic lateral sclerosis
ApoE	Apolipoprotein E
ASC	Apoptosis-associated speck-like protein containing a CARD
ATP	Adenosine triphosphate
C9orf72	Chromosome 9 open reading frame 72
cGAS	Cyclic GMP-AMP synthase
CNS	Central nervous system
CSF	Cerebrospinal fluid
DAM	Disease-associated microglia
DAMPs	Damage-associated molecular patterns
DAP12	DNAX-activation protein10/12
DPRs	Dipeptide repeat proteins
DSBs	DNA double-strand breaks
dsDNA	Double-stranded DNA
ERGIC	ER-Golgi intermediate compartment
FTD	Frontotemporal dementia
FUS	Fused in sarcoma
GSDMD	Gasdermin D
IFN-I	Type I interferon
IL-1β	Interleukin-1β
ISGs	Interferon-stimulated genes
mPTP	Mitochondrial permeability transition pore
mtDNA	Mitochondrial DNA
NF-κB	Nuclear factor-κB
NLRP3	NOD-, LRR- and pyrin domain-containing protein 3
PAMPs	Pathogen-associated molecular patterns
PERK	PKR-like endoplasmic reticulum kinase
poly-GA	Poly-Gly-Ala
PRRs	Pattern recognition receptors
PTMs	Post translational modifications
ROS	Reactive oxygen species
SOD1	Superoxide dismutase 1
STING	Stimulator of interferon genes
sTREM2	Soluble TREM2
TAK1	Transforming growth factor-β-activated kinase 1
TBK1	TANK binding kinase 1
TDP-43	Transactive response DNA-binding protein 43
TFEB	Transcription factor EB
TREM2	Triggering receptor expressed on myeloid cells 2
VDAC	Voltage-dependent anion channel

## References

[bib1] Baizabal-Carvallo J.F., Jankovic J. (2016). Parkinsonism, movement disorders and genetics in frontotemporal dementia. Nature Reviews Neurology.

[bib2] Balendra R., Isaacs A.M. (2018). C9orf72-mediated ALS and FTD: Multiple pathways to disease. Nature Reviews Neurology.

[bib3] Balka K.R. (2020). TBK1 and IKKepsilon act redundantly to mediate STING-induced NF-kappaB responses in myeloid cells. Cell Reports.

[bib4] Bellezza I. (2018). Peroxynitrite activates the NLRP3 inflammasome Cascade in SOD1(G93A) mouse model of amyotrophic lateral sclerosis. Molecular Neurobiology.

[bib5] Borroni B. (2014). Heterozygous TREM2 mutations in frontotemporal dementia. Neurobiology of Aging.

[bib6] Brenner D. (2019). Heterozygous Tbk1 loss has opposing effects in early and late stages of ALS in mice. Journal of Experimental Medicine.

[bib7] Broz P., Dixit V.M. (2016). Inflammasomes: Mechanism of assembly, regulation and signalling. Nature Reviews Immunology.

[bib8] Cady J. (2014). TREM2 variant p.R47H as a risk factor for sporadic amyotrophic lateral sclerosis. JAMA Neurology.

[bib9] Carling G.K. (2024). Alzheimer's disease-linked risk alleles elevate microglial cGAS-associated senescence and neurodegeneration in a tauopathy model. Neuron.

[bib10] Chen X. (2015). Assessment of TREM2 rs75932628 association with amyotrophic lateral sclerosis in a Chinese population. Journal of the Neurological Sciences.

[bib11] Chen C., Xu P. (2023). Cellular functions of cGAS-STING signaling. Trends in Cell Biology.

[bib12] Chen T., Zhang W., Huang B., Chen X., Huang C. (2020). UBQLN2 promotes the production of type I interferon via the TBK1-IRF3 pathway. Cells.

[bib13] Cihankaya H. (2024). Elevated NLRP3 inflammasome activation is associated with motor neuron degeneration in ALS. Cells.

[bib14] Clenet M.L. (2023). Divergent functional outcomes of NLRP3 blockade downstream of multi-inflammasome activation: Therapeutic implications for ALS. Frontiers in Immunology.

[bib15] Coll R.C. (2015). A small-molecule inhibitor of the NLRP3 inflammasome for the treatment of inflammatory diseases. Natura Med.

[bib16] Coll R.C. (2019). MCC950 directly targets the NLRP3 ATP-Hydrolysis motif for inflammasome inhibition. Nature Chemical Biology.

[bib17] Cooper-Knock J. (2017). A data-driven approach links microglia to pathology and prognosis in amyotrophic lateral sclerosis. Acta Neuropathologica Communications.

[bib18] Cuyvers E. (2014). Investigating the role of rare heterozygous TREM2 variants in Alzheimer's disease and frontotemporal dementia. Neurobiology of Aging.

[bib19] Debye B. (2018). Neurodegeneration and NLRP3 inflammasome expression in the anterior thalamus of SOD1(G93A) ALS mice. Brain Pathology.

[bib20] Deczkowska A., Weiner A., Amit I. (2020). The physiology, pathology, and potential therapeutic applications of the TREM2 signaling pathway. Cell.

[bib21] Deora V. (2020). The microglial NLRP3 inflammasome is activated by amyotrophic lateral sclerosis proteins. Glia.

[bib22] Dezfouli M.A. (2025). Circulating miR-223/NLRP3 axis and IL-1beta level in functional disease progression of amyotrophic lateral sclerosis. Acta Neurologica Belgica.

[bib23] Dols-Icardo O. (2020). Motor cortex transcriptome reveals microglial key events in amyotrophic lateral sclerosis. Neurol Neuroimmunol Neuroinflamm.

[bib24] Feldman E.L. (2022). Amyotrophic lateral sclerosis. Lancet.

[bib25] Feuerbach D. (2017). ADAM17 is the main sheddase for the generation of human triggering receptor expressed in myeloid cells (hTREM2) ectodomain and cleaves TREM2 after Histidine 157. Neuroscience Letters.

[bib26] Freibaum B.D. (2015). GGGGCC repeat expansion in C9orf72 compromises nucleocytoplasmic transport. Nature.

[bib27] Freischmidt A. (2015). Haploinsufficiency of TBK1 causes familial ALS and fronto-temporal dementia. Nature Neuroscience.

[bib28] Fu R.H. (2022). C9-ALS-Associated proline-arginine dipeptide repeat protein induces activation of NLRP3 inflammasome of HMC3 microglia cells by binding of complement component 1 Q subcomponent-binding protein (C1QBP), and syringin prevents this effect. Cells.

[bib29] Giannoccaro M.P. (2017). Multiple variants in families with amyotrophic lateral sclerosis and frontotemporal dementia related to C9orf72 repeat expansion: Further observations on their oligogenic nature. Journal of Neurology.

[bib30] Giraldo M. (2013). Variants in triggering receptor expressed on myeloid cells 2 are associated with both behavioral variant frontotemporal lobar degeneration and Alzheimer's disease. Neurobiology of Aging.

[bib31] Guerreiro R.J. (2013). Using exome sequencing to reveal mutations in TREM2 presenting as a frontotemporal dementia-like syndrome without bone involvement. JAMA Neurology.

[bib32] Guerreiro R. (2013). Novel compound heterozygous mutation in TREM2 found in a Turkish frontotemporal dementia-like family. Neurobiology of Aging.

[bib33] Gui X. (2019). Autophagy induction via STING trafficking is a primordial function of the cGAS pathway. Nature.

[bib34] Guven G. (2026). Increased plasma soluble TREM2 levels in non-alzheimer's dementia. Acta Neurologica Belgica.

[bib35] Heitzer M. (2017). Administration of 17beta-Estradiol improves motoneuron survival and down-regulates inflammasome activation in Male SOD1(G93A) ALS mice. Molecular Neurobiology.

[bib36] Henkel J.S., Beers D.R., Zhao W., Appel S.H. (2009). Microglia in ALS: The good, the bad, and the resting. Journal of Neuroimmune Pharmacology.

[bib37] Hiew J.Y., Lim Y.S., Liu H., Ng C.S. (2025). Integrated transcriptomic profiling reveals a STING-mediated Type II interferon signature in SOD1-mutant amyotrophic lateral sclerosis models. Communications Biology.

[bib38] Hopfner K.P., Hornung V. (2020). Molecular mechanisms and cellular functions of cGAS-STING signalling. Nature Reviews Molecular Cell Biology.

[bib39] Hornung V. (2008). Silica crystals and aluminum salts activate the NALP3 inflammasome through phagosomal destabilization. Nature Immunology.

[bib40] Ishikawa H., Barber G.N. (2008). STING is an endoplasmic reticulum adaptor that facilitates innate immune signalling. Nature.

[bib41] Italiani P. (2014). Evaluating the levels of interleukin-1 family cytokines in sporadic amyotrophic lateral sclerosis. Journal of Neuroinflammation.

[bib42] Jericó I. (2023). Profiling TREM2 expression in amyotrophic lateral sclerosis. Brain, Behavior, and Immunity.

[bib43] Jiang H. (2019). Chromatin-bound cGAS is an inhibitor of DNA repair and hence accelerates genome destabilization and cell death. EMBO Journal.

[bib44] Jiang X. (2026). Blocking RAN translation without altering repeat RNAs rescues C9ORF72-related ALS and FTD phenotypes. Science.

[bib45] Jiao L. (2024). sTREM2 cerebrospinal fluid levels are a potential biomarker in amyotrophic lateral sclerosis and associate with UMN burden. Frontiers in Neurology.

[bib46] Johann S. (2015). NLRP3 inflammasome is expressed by astrocytes in the SOD1 mouse model of ALS and in human sporadic ALS patients. Glia.

[bib47] Kayagaki N. (2015). Caspase-11 cleaves gasdermin D for non-canonical inflammasome signalling. Nature.

[bib48] Keren-Shaul H. (2017). A unique microglia type associated with restricting development of Alzheimer's Disease. Cell.

[bib49] Kleinberger G. (2014). TREM2 mutations implicated in neurodegeneration impair cell surface transport and phagocytosis. Science Translational Medicine.

[bib50] Kleinberger G. (2017). The FTD-Like syndrome causing TREM2 T66M mutation impairs microglia function, brain perfusion, and glucose metabolism. EMBO Journal.

[bib51] Krasemann S. (2017). The TREM2-APOE pathway drives the transcriptional phenotype of dysfunctional microglia in neurodegenerative diseases. Immunity.

[bib53] Lall D., Baloh R.H. (2017). Microglia and C9orf72 in neuroinflammation and ALS and frontotemporal dementia. Journal of Clinical Investigation.

[bib54] Lattante S. (2013). TREM2 mutations are rare in a French cohort of patients with frontotemporal dementia. Neurobiology of Aging.

[bib55] Le Ber I. (2014). Homozygous TREM2 mutation in a family with atypical frontotemporal dementia. Neurobiology of Aging.

[bib56] Lee K.H. (2016). C9orf72 dipeptide repeats impair the assembly, dynamics, and function of membrane-less organelles. Cell.

[bib57] Lill C.M. (2015). The role of TREM2 R47H as a risk factor for Alzheimer's disease, frontotemporal lobar degeneration, amyotrophic lateral sclerosis, and Parkinson's disease. Alzheimer's & Dementia.

[bib58] Ling S.C., Polymenidou M., Cleveland D.W. (2013). Converging mechanisms in ALS and FTD: Disrupted RNA and protein homeostasis. Neuron.

[bib59] Liu S. (2015). Phosphorylation of innate immune adaptor proteins MAVS, STING, and TRIF induces IRF3 activation. Science.

[bib60] Liu H. (2018). Nuclear cGAS suppresses DNA repair and promotes tumorigenesis. Nature.

[bib61] Liu S., Wang A., Chen C., Xu P. (2025). Organelle-specific signaling of cGAS-STING. Trends in Cell Biology.

[bib62] Liu Y., Xu P. (2025). cGAS, an innate dsDNA sensor with multifaceted functions. Cell Insight.

[bib63] Lu A. (2014). Unified polymerization mechanism for the assembly of ASC-dependent inflammasomes. Cell.

[bib64] Lv B. (2024). A TBK1-independent primordial function of STING in lysosomal biogenesis. Molecular Cell.

[bib65] Ma M., Jiang W., Zhou R. (2024). DAMPs and DAMP-Sensing receptors in inflammation and diseases. Immunity.

[bib66] Maier A. (2015). Interleukin-1 antagonist anakinra in amyotrophic lateral Sclerosis--A pilot study. PLoS One.

[bib67] Mallika A.P., Mathuranath P.S., Menon R.N., Banerjee M. (2026). A novel rare homozygous R47C variant in TREM2 with frontal variant Alzheimer's disease. Neurology India.

[bib68] Marques C. (2024). Neuronal STING activation in amyotrophic lateral sclerosis and frontotemporal dementia. Acta Neuropathologica.

[bib69] McCauley M.E., Baloh R.H. (2019). Inflammation in ALS/FTD pathogenesis. Acta Neuropathologica.

[bib70] McCauley M.E. (2020). C9orf72 in myeloid cells suppresses STING-Induced inflammation. Nature.

[bib71] Meissner F., Molawi K., Zychlinsky A. (2010). Mutant superoxide dismutase 1-induced IL-1beta accelerates ALS pathogenesis. Proceedings of the National Academy of Sciences of the United States of America.

[bib72] Mitra J. (2019). Motor neuron disease-associated loss of nuclear TDP-43 is linked to DNA double-strand break repair defects. Proceedings of the National Academy of Sciences of the United States of America.

[bib73] Moreno-Garcia L. (2021). Inflammasome in ALS skeletal muscle: NLRP3 as a potential biomarker. International Journal of Molecular Sciences.

[bib74] Mukai K. (2016). Activation of STING requires palmitoylation at the Golgi. Nature Communications.

[bib75] Munoz-Planillo R. (2013). K(+) efflux is the common trigger of NLRP3 inflammasome activation by bacterial toxins and particulate matter. Immunity.

[bib76] Neumann M. (2006). Ubiquitinated TDP-43 in frontotemporal lobar degeneration and amyotrophic lateral sclerosis. Science.

[bib77] Ng A.S.L. (2018). Targeted exome sequencing reveals homozygous TREM2 R47C mutation presenting with behavioral variant frontotemporal dementia without bone involvement. Neurobiology of Aging.

[bib78] Ng A.S., Rademakers R., Miller B.L. (2015). Frontotemporal dementia: A bridge between dementia and neuromuscular disease. Annals of the New York Academy of Sciences.

[bib79] Nguyen M. (2025). MAPL regulates gasdermin-mediated release of mtDNA from lysosomes to drive pyroptotic cell death. Nature Cell Biology.

[bib80] Ogonowski N. (2023). Frontotemporal dementia presentation in patients with heterozygous p.H157Y variant of TREM2. Journal of Medical Genetics.

[bib81] Peplonska B. (2018). TREM2 variants in neurodegenerative disorders in the Polish population. Homozygosity and compound heterozygosity in FTD patients. Amyotroph Lateral Scler Frontotemporal Degener.

[bib82] Poddar S. (2025). ArfGAP2 promotes STING proton channel activity, cytokine transit, and autoinflammation. Cell.

[bib83] Rauchmann B.S., Sadlon A., Perneczky R. (2020). Soluble TREM2 and inflammatory proteins in Alzheimer's disease cerebrospinal fluid. Journal of Alzheimer's Disease.

[bib84] Rayaprolu S. (2013). TREM2 in neurodegeneration: Evidence for association of the p.R47H variant with frontotemporal dementia and Parkinson's disease. Molecular Neurodegeneration.

[bib85] Renton A.E. (2011). A hexanucleotide repeat expansion in C9ORF72 is the cause of chromosome 9p21-linked ALS-FTD. Neuron.

[bib86] Ruiz A. (2014). Assessing the role of the TREM2 p.R47H variant as a risk factor for Alzheimer's disease and frontotemporal dementia. Neurobiology of Aging.

[bib87] Samanci B. (2021). TREM2 variants as a possible cause of frontotemporal dementia with distinct neuroimaging features. European Journal of Neurology.

[bib88] Shang G., Zhang C., Chen Z.J., Bai X.C., Zhang X. (2019). Cryo-EM structures of STING reveal its mechanism of activation by cyclic GMP-AMP. Nature.

[bib89] Shao W. (2022). Two FTD-ALS genes converge on the endosomal pathway to induce TDP-43 pathology and degeneration. Science.

[bib90] Shu X. (2023). Negative regulation of TREM2-mediated C9orf72 poly-GA clearance by the NLRP3 inflammasome. Cell Reports.

[bib91] Shu X., Zeng J., Zhang K. (2026). TREM2-mediated crosstalk in ALS: Microglial fate transition, protein aggregate clearance, and peripheral nerve repair. Neuroscience Bulletin.

[bib92] Siokas V. (2021). Lack of association between TREM2 rs75932628 variant and amyotrophic lateral sclerosis. Molecular Biology Reports.

[bib93] Slattery C.F. (2014). R47H TREM2 variant increases risk of typical early-onset Alzheimer's disease but not of prion or frontotemporal dementia. Alzheimer's & Dementia.

[bib94] Spiller K.J. (2018). Microglia-mediated recovery from ALS-relevant motor neuron degeneration in a mouse model of TDP-43 proteinopathy. Nature Neuroscience.

[bib95] Strong M.J. (2009). Consensus criteria for the diagnosis of frontotemporal cognitive and behavioural syndromes in amyotrophic lateral sclerosis. Amyotrophic Lateral Sclerosis.

[bib96] Suárez-Calvet M. (2016). sTREM2 cerebrospinal fluid levels are a potential biomarker for microglia activity in early-stage Alzheimer's disease and associate with neuronal injury markers. EMBO Molecular Medicine.

[bib97] Sun W. (2009). ERIS, an endoplasmic reticulum IFN stimulator, activates innate immune signaling through dimerization. Proceedings of the National Academy of Sciences of the United States of America.

[bib98] Sun L., Wu J., Du F., Chen X., Chen Z.J. (2013). Cyclic GMP-AMP synthase is a cytosolic DNA sensor that activates the type I interferon pathway. Science.

[bib99] Swanson K.V., Deng M., Ting J.P. (2019). The NLRP3 inflammasome: Molecular activation and regulation to therapeutics. Nature Reviews Immunology.

[bib100] Tan H.Y. (2022). cGAS and DDX41-STING mediated intrinsic immunity spreads intercellularly to promote neuroinflammation in SOD1 ALS model. iScience.

[bib101] Thelen M. (2014). Investigation of the role of rare TREM2 variants in frontotemporal dementia subtypes. Neurobiology of Aging.

[bib102] Tsuchida T. (2010). The ubiquitin ligase TRIM56 regulates innate immune responses to intracellular double-stranded DNA. Immunity.

[bib103] Udeochu J.C. (2023). Tau activation of microglial cGAS-IFN reduces MEF2C-mediated cognitive resilience. Nature Neuroscience.

[bib104] Ulland T.K., Colonna M. (2018). TREM2 - A key player in microglial biology and Alzheimer disease. Nature Reviews Neurology.

[bib105] van der Ende E.L. (2021). CSF sTREM2 is elevated in a subset in GRN-related frontotemporal dementia. Neurobiology of Aging.

[bib106] Wang S. (2022). TREM2 drives microglia response to amyloid-β via SYK-dependent and -independent pathways. Cell.

[bib107] Wang A. (2024). Innate immune sensing of lysosomal dysfunction drives multiple lysosomal storage disorders. Nature Cell Biology.

[bib108] Wang L. (2025). TREM2 affects DAM-Like cell transformation in the acute phase of TBI in mice by regulating microglial glycolysis. Journal of Neuroinflammation.

[bib109] Wang R., Yang B., Zhang D. (2011). Activation of interferon signaling pathways in spinal cord astrocytes from an ALS mouse model. Glia.

[bib110] Wood A., Gurfinkel Y., Polain N., Lamont W., Lyn Rea S. (2021). Molecular mechanisms underlying TDP-43 pathology in cellular and animal models of ALS and FTLD. International Journal of Molecular Sciences.

[bib111] Woollacott I.O.C. (2022). CSF glial markers are elevated in a subset of patients with genetic frontotemporal dementia. Annals of Clinical and Translational Neurology.

[bib112] Wunderlich P. (2013). Sequential proteolytic processing of the triggering receptor expressed on myeloid cells-2 (TREM2) protein by ectodomain shedding and γ-secretase-dependent intramembranous cleavage. Journal of Biological Chemistry.

[bib113] Xian H. (2022). Oxidized DNA fragments exit mitochondria via mPTP- and VDAC-Dependent channels to activate NLRP3 inflammasome and interferon signaling. Immunity.

[bib114] Xiao Q. (2007). Mutant SOD1(G93A) microglia are more neurotoxic relative to wild-type microglia. Journal of Neurochemistry.

[bib115] Xie M. (2022). TREM2 interacts with TDP-43 and mediates microglial neuroprotection against TDP-43-related neurodegeneration. Nature Neuroscience.

[bib116] Xie M. (2025). Rod-shaped microglia interact with neuronal dendrites to attenuate cortical excitability during TDP-43-related neurodegeneration. Immunity.

[bib117] Xun J. (2024). A conserved ion channel function of STING mediates noncanonical autophagy and cell death. EMBO Reports.

[bib118] Yeh F.L., Hansen D.V., Sheng M. (2017). TREM2, Microglia, and neurodegenerative diseases. Trends in Molecular Medicine.

[bib119] Yu C.H. (2020). TDP-43 triggers mitochondrial DNA release via mPTP to activate cGAS/STING in ALS. Cell.

[bib120] Zhang Y.J. (2018). Poly(GR) impairs protein translation and stress granule dynamics in C9orf72-associated frontotemporal dementia and amyotrophic lateral sclerosis. Natura Med.

[bib121] Zhang C. (2019). Structural basis of STING binding with and phosphorylation by TBK1. Nature.

[bib122] Zhang K. (2021). UBQLN2-HSP70 axis reduces poly-gly-ala aggregates and alleviates behavioral defects in the C9ORF72 animal model. Neuron.

[bib123] Zhang D. (2022). A non-canonical cGAS-STING-PERK pathway facilitates the translational program critical for senescence and organ fibrosis. Nature Cell Biology.

[bib124] Zhang H. (2022). Spatiotemporal evolution of pyroptosis and canonical inflammasome pathway in hSOD1(G93A) ALS mouse model. BMC Neuroscience.

[bib125] Zhang W. (2022). Cytosolic escape of mitochondrial DNA triggers cGAS-STING-NLRP3 axis-dependent nucleus pulposus cell pyroptosis. Experimental and Molecular Medicine.

[bib126] Zhang B., Li R., Zhang Y., Gao X. (2020). Differential role of triggering receptors expressed on myeloid cells 2 R47H in 3 neurodegenerative diseases based on a systematic review and meta-analysis. Medicine (Baltimore).

[bib127] Zhao W. (2010). Extracellular mutant SOD1 induces microglial-mediated motoneuron injury. Glia.

[bib128] Zhao W. (2015). TDP-43 activates microglia through NF-kappaB and NLRP3 inflammasome. Experimental Neurology.

[bib129] Zhao B. (2020). The molecular basis of tight nuclear tethering and inactivation of cGAS. Nature.

[bib130] Zhong B. (2008). The adaptor protein MITA links virus-sensing receptors to IRF3 transcription factor activation. Immunity.

[bib131] Zhong B. (2009). The ubiquitin ligase RNF5 regulates antiviral responses by mediating degradation of the adaptor protein MITA. Immunity.

[bib132] Zhong L. (2017). Soluble TREM2 induces inflammatory responses and enhances microglial survival. Journal of Experimental Medicine.

[bib133] Zhong L. (2019). Soluble TREM2 ameliorates pathological phenotypes by modulating microglial functions in an Alzheimer's disease model. Nature Communications.

[bib134] Zhou S.L. (2019). TREM2 variants and neurodegenerative diseases: A systematic review and meta-analysis. Journal of Alzheimer's Disease.

